# Investigating *InDels* in *YAP* and *TAZ* genes and their impact on growth characteristics in goats

**DOI:** 10.5194/aab-67-343-2024

**Published:** 2024-07-09

**Authors:** Wanxia Zhao, Ziteng Wang, Yichen Lei, Xiaoqin Tang, Xiaohua Yi, Junyi Jiang, Jiapeng Li, Shuhui Wang, Xiuzhu Sun

**Affiliations:** 1 College of Animal Science and Technology, Northwest A&F University, Yangling, Shaanxi 712100, PR China; 2 College of Grassland Agriculture, Northwest A&F University, Yangling, Shaanxi 712100, PR China

## Abstract

Yes-associated protein (*YAP*) and a transcriptional co-activator with PDZ-binding motif (*TAZ*) genes are crucial for regulating the size of mammalian tissues and organs as well as for many biological processes such as bone formation, cell lineage determination, tissue regeneration, and cell proliferation. The purpose of this study was to characterize the *YAP* and *TAZ* gene polymorphisms in 266 Guanzhong Dairy Goats and 299 Shanbei White Cashmere Goats and to explore their potential relationship with growth characteristics such as body weight and body length. After genotyping and using PCR amplification and Sanger sequencing to find polymorphisms in the *YAP* and *TAZ* genes, five *InDels* loci were found in the goat *YAP* gene and three *InDels* loci in the *TAZ* gene. The findings of the association analysis demonstrated that the goats' body weight, height, cannon circumference, chest depth, chest breadth, and chest circumference were all substantially influenced by five *InDels* loci in the *YAP* gene ( p<0.05 ). Goat body height, trunk breadth, trunk length, body length, and body weight were all substantially impacted by three *InDels* loci in the *TAZ* gene ( p<0.05 ). In conclusion, eight *InDels* loci of goat *YAP* and *TAZ* were found in this study, and their impacts on goat phenotype were disclosed. These results might offer fresh avenues for boosting goat molecular breeding.

## 1 Introduction

The transcriptional co-activator with a PDZ-binding motif (*TAZ*) and a yes-associated protein (*YAP*) are downstream effectors of the Hippo signaling pathway. *TAZ* and *YAP* have a 46 % amino acid sequence identity in mammals, and they can interact with transcription factors that contain the PPXY motif via the WW structural domain (Pocaterra et al., 2020). *YAP* and *TAZ* have garnered significant interest in recent times due to their vital functions in the formation and maintenance of tissue homeostasis in organisms. The activity of *YAP* and *TAZ *is essential for tissue-specific progenitor cell growth and cell proliferation during tissue renewal and regeneration (Heng et al., 2021; Piccolo et al., 2014). *YAP* and *TAZ* are involved in signaling during osteogenesis and play a critical role in the formation of bone and cartilage by modulating regulatory factors such as Runx2, Osx, and Sox9. The *YAP* and *TAZ* genes were knocked down using Prx1-Creo and Ose-Cre (Zarka et al., 2022; Xiong et al., 2018). As a result, mice experienced miscarriages during the perinatal stage, and the embryos displayed thoracic bleeding and skeletal abnormalities. Following *TAZ *gene knockdown using Col2-Cre, Y. Li et al. (2021) observed that young mouse pups showed growth retardation and underdevelopment of the sternum, ribs, and skull. In addition, it has been shown that the *YAP* gene plays an important role in muscle development in sheep and New Zealand white rabbits, regulating related genes such as *MSTN*, *MYH6*, and *MYOD* and other related genes associated with growth traits in livestock and poultry (Liu et al., 2023). Moreover, the *YAP* gene is an important pathway associated with susceptibility to brucellosis in sheep as well as a related candidate gene. As a result, *YAP* and *TAZ* contribute to bone production and are essential for animals' appropriate growth and development (X. Li et al., 2021).

According to Matin and Nadeau (2001), growth traits are quantitative traits regulated by microevolution polygenes that are impacted by both heredity and environment. Furthermore, more precise and efficient procedures are required for the selection of growth features. Animal breeding frequently employs molecular marker-assisted selection methods (Yan et al., 2014; Lin et al., 2021). One of the primary causes of molecular diversity among them is insertion/deletion. *InDels* are commonly employed in the research of the growth and economic aspects of domestic animals. It is essential in both genetic diversity and phenotypic differentiation (Cui et al., 2018). In light of the critical role that *YAP/TAZ* plays in growth and development, we examined the *InDels* of the *YAP/TAZ* gene in Guanzhong Dairy Goat and Shanbei White Cashmere Goat, assessing their effects on goat growth and development in order to identify key marker genes and furnish fundamental information that will expedite goat molecular breeding.

## 2 Materials and methods

### 2.1 Sample collection and data collection

A total of 299 Shanbei White Cashmere Goats (SWCGs, Fugu, Shaanxi) and 266 Guanzhong Dairy Goats (GZDGs, Fuping, Shaanxi) were among the 565 local goats, ages 1–4 years, who were randomly chosen. Throughout the whole experiment, the management and feeding circumstances remained constant. All the goats' venous blood was drawn, and the growth characteristics were noted. The primary data consisted of the following: body height (BH), height at hip cross (HHC), body length (BL), rump length (RL), rump height (RH), rump width (RW), trunk length (TL), waist angle width (WAW), chest width (ChW), chest depth (ChD), chest circumference (ChC), cannon circumference (CaC), and body weight (BW). A single responsible individual measured each growth attribute to reduce measurement errors.

### 2.2 Genomic DNA extraction and DNA mixing pool construction

A Nanodrop1000 Spectrophotometer (Thermo Science, Waltham, MA, USA) was used to measure the concentration and purity (A260/280) of the extracted genomic DNA. The whole blood genomic DNA fast-extraction kit was kept at -80 °C. After diluting each sample to 20 ng µ L ${}^{-1}$ with ddH ${}_{2}$ O, it was kept at -20 °C. To identify putative *InDels* loci in the goat *YAP/TAZ* gene, 30 DNA samples were chosen at random from each breed and combined to create two DNA pools. 

### 2.3 *InDels* loci discovery and primer design

Five potential *InDels* loci were found in the goat *YAP* (GenBank accession no. NC_030822.1) gene, and three potential *InDels* loci were found in the goat *TAZ* (GenBank accession no. NC_030808.1) gene, according to the Ensemble database (http://asia.ensembl.org/index.html, last access: 20 May 2022) and the GGVD database (http://animal.nwsuaf.edu.cn/code/index.php/GoatVar, last access: 25 July 2022) (Fu et al., 2021). Primer Premier Software 5.0 was used to design the primers (Table 1).

**Table 1:** Primers and related site information for PCR amplification of the *YAP/TAZ* gene.

Primer name	Primer sequence (5 ${}^{\prime }$ –3 ${}^{\prime }$ )	Product size (bp)	*InDels* sequence	Variant ID
YAP-InDel-1	F:TTCCTATCTCAACTTTGGTA	144/153	ins ATAATTTGA	Novel
	R:TGAAATGTTAAGAAGCACTC			
YAP-InDel-2	F:GTCAAATACCTTTTGAAACT	140/151	ins AATTTCTTTTC	Novel
	R:ATGTCCTTGTAACTCACCTA			
YAP-InDel-3	F:ACACGGTGCTTAGACTTGGA	156/150	del TTTCCG	Novel
	R:GACGCTAAAATGACACTCCC			
YAP-InDel-4	F:TGATGAACTTAAAACCAT	171/154	del CACATGAGAGCTCATTT	rs653744801
	R:TGCAGTGCTCTTACACTA			
YAP-InDel-5	F:TGGACTGCCAACACACTCAG	191/208	ins AGTATTGTACTGTAATA	rs645491525
	R:TTCAGGGTACTATACTGCAAGGT			
TAZ-InDel-6	F:CATAACCGTAGGTGCTTT	129/122	del TTCTTTC	rs643491369
	R:CTGGGCCATGTACTCAAC			
TAZ-InDel-7	F:GGAGCAAGGGTTCAATCT	161/175	ins CATGCCGTTTCCCC	rs670885411
	R:TCCAATATCACAAGTCCC			
TAZ-InDel-8	F:TCAGGACTAAGTTGGAGA	171/178	ins AAAAACA	rs665371805
	R:TCTTGGGAAAACGACACT			

### 2.4 Genotyping and DNA sequencing

The goat *YAP/TAZ* gene's *InDels* polymorphism was genotyped after the target gene was amplified using touch-down PCR, and the amplified products were separated by 3 % agarose gel electrophoresis. Generally speaking, genotypes were classified as homozygous (insertion/deletion: II), heterozygous (insertion/deletion: ID), and homozygous (deletion/deletion: DD). The *YAP/TAZ* gene mutation was confirmed by sequencing the PCR results of various genotypes.

### 2.5 Statistical analysis

Using the Botstein et al. (1980) approach, the genotype and allele frequencies of *InDels* mutations in goat breeds were determined. Using Haploview 4.2 software, linkage disequilibrium analysis of haplotypes at the *InDels* locus of the *YAP/TAZ* gene was carried out concurrently in two local breeds of sheep (Barrett, 2009). Utilizing Nei's (Nei and Roychoudhury, 1974; Nei and Li, 1979) methodology, the population's genetic diversity index was determined. The population's genetic variation was measured using the homozygosity (Ho), heterozygosity (He), and polymorphism information content (PIC), which served as an index of population polymorphism.

The statistical linear model is $Y_{ijk}=\mu +A_{i}+B_{j}+G_{k}+$ ${}_{ijk}$ , where $Y_{ijk}$ is the observation of growth-related traits, μ is the overall mean for each trait, $A_{i}$ is the effect of the i th age, B ${}_{j}$ is the effect of the i th sex, $G_{k}$ is the fixed effect of the genotype or combined genotype, and $E_{ijk}$ is a random residual. The fixed effect of genotypes was the main reason for the difference in growth traits. When the p value was less than 0.05, the difference was considered significant. Using GraphPad Prism 9 (GraphPad Software, USA) software, the Hardy–Weinberg equilibrium (HWE) state was analyzed using a chi-squared test ( $\chi^{2}$ test). The correlation between the *YAP/TAZ* gene and growth traits of goats was analyzed by one-way ANOVA using SPSS 24 (IBM, USA) software. 

## 3 Results

### 3.1 Identification of genetic variants in the goats' *YAP/TAZ* gene

**Figure 1:**
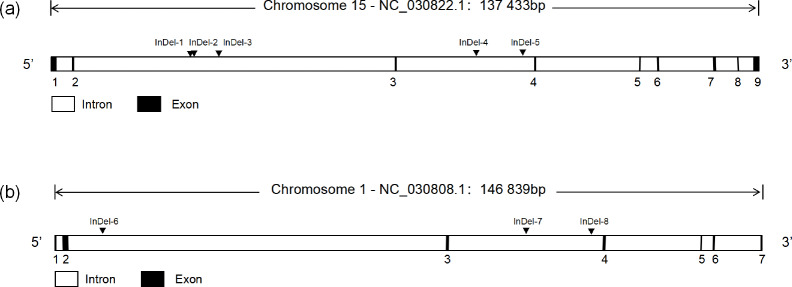
The structure of the goat *YAP/TAZ* gene and the location of *InDels* mutation. **(a)** *YAP* gene structure map and *InDels* locus. **(b)** *TAZ* gene structure map and *InDels* locus.

**Figure 2:**
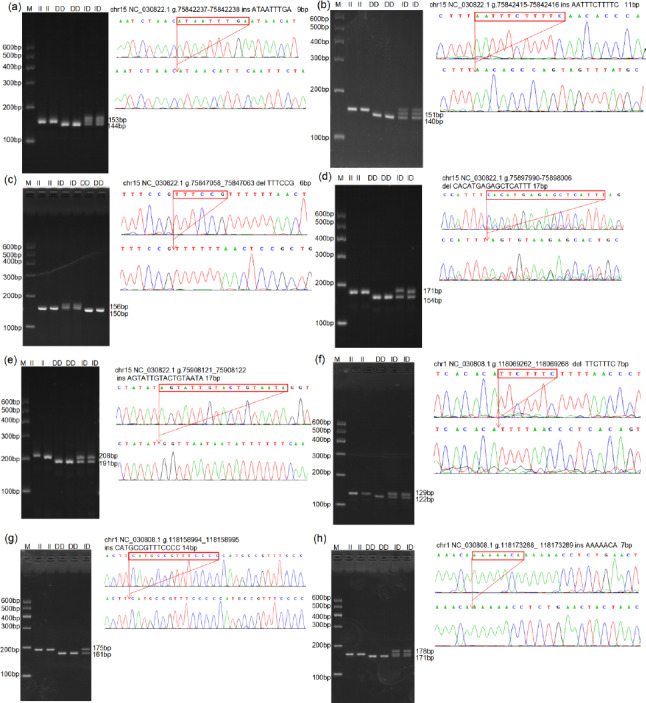
Agarose gel map and sequencing map of the goat *YAP/TAZ* gene *InDels* mutation site. Panels **(a)**–**(h)** show the agarose gel and sequencing maps of InDel-1 to InDel-8.

Through the analysis of DNA sequencing data, three *InDels* mutation sites (InDel-1 to InDel-8) were found in the *TAZ *gene's intron region, and five *InDels* mutation sites (InDel-1 to InDel-8) were found in the *YAP* gene's intron region (Fig. 1). By using 3 % agarose gel electrophoresis and sequencing, the genotyping of the *InDels* mutation sites of the *YAP/TAZ* gene was demonstrated (Fig. 2). Shanbei White Cashmere Goat is the only one with the following *InDels* loci: InDel-3 (NC_030822.1 g.75847058_75847063 del TTTCCG), InDel-4 (rs653744801, del CACATGAGAGCTCATTT), InDel-5 (rs645491525, ins AGTATTGTACTGTAATA), and InDel-6 (rs643491369, del TTCTTTC). Guanzhong Dairy Goat is the only one with InDel-2 (NC_030822.1 g.75842415-75842416 ins AATTTCTTTTC) and InDel-7 (rs670885411, ins CATGCCGTTTCCCC) (Fig. 2b, g). Both goat breeds have the InDel-1 (NC_030822.1 g.75842237-75842238 ins ATAATTTGA) and InDel-8 (rs665371805, ins AAAAACA) loci (Fig. 2a, h). It is important to note that InDel-1, InDel-2, and InDel-3 are unique in the GGVD database but not in the Ensemble database.

### 3.2 Analysis of the genetic polymorphisms

The genotype frequencies, allele frequencies, and polymorphism data of eight *InDels* loci of the *YAP/TAZ* gene were computed and examined in order to comprehend the variation of the *YAP/TAZ* gene in goats. It was discovered that the dominant genotypes in InDel-1, InDel-3, and InDel-6 were type II. In InDel-4 and InDel-8 it was ID, and in InDel-5 it was DD. In Shanbei White Cashmere Goat, the I allele frequencies of five *InDels* loci varied from 0.319 to 0.923, whereas the D allele frequencies varied from 0.077 to 0.681 (Table 2). The dominant genotype of InDel-1 and InDel-2 in Guanzhong Dairy Goat was the DD genotype, the dominant allele of InDel-7 was the ID genotype, and the dominant genotype of InDel-8 was the II genotype. Table 2 shows that the D allele frequencies of the four *InDels* loci ranged from 0.318 to 0.703, whereas the I allele rates fell between 0.297 and 0.682. The two goats had a genetic heterozygosity of 0.142 to 0.500 and an effective allele count of 1.166 to 1.999 (Table 3). The remaining *InDels* loci are in the moderate polymorphism range ( 0.25< PIC <0.5 ), with the exception of the Shanbei White Cashmere Goat InDel-1, which is in the low polymorphism range (PIC <0.25 ) (Table 3). Furthermore, the Hardy–Weinberg equilibrium of Guanzhong Dairy Goat and Shanbei White Cashmere Goat was confirmed using the $\chi^{2}$ test, and all of them were in equilibrium ( p>0.05 ) (Table 2).

**Table 2:** Genotype and gene frequencies of the *InDels* locus in the *YAP/TAZ* gene.

Variety	Site	Number	Genotype and gene frequency					Hardy–Weinberg	
								equilibrium	
			II	ID	DD	I	D	$\chi^{2}$	p
SWCG	InDel-1	299	0.846	0.154	0.000	0.923	0.077	2.190	0.335
	InDel-3	299	0.605	0.368	0.027	0.789	0.211	1.836	0.399
	InDel-4	299	0.308	0.538	0.154	0.577	0.423	1.705	0.426
	InDel-5	299	0.097	0.445	0.458	0.319	0.681	0.114	0.945
	InDel-6	299	0.579	0.371	0.050	0.764	0.236	0.176	0.916
	InDel-8	299	0.244	0.495	0.261	0.492	0.508	0.015	0.993
GZDG	InDel-1	266	0.105	0.383	0.511	0.297	0.703	0.962	0.618
	InDel-2	266	0.316	0.470	0.214	0.551	0.449	0.324	0.850
	InDel-7	266	0.226	0.511	0.263	0.481	0.519	0.093	0.955
	InDel-8	266	0.503	0.357	0.140	0.682	0.318	4.332	0.115

SWCG: Shanbei White Cashmere Goat; GZDG: Guanzhong Dairy Goat; II: insertion/insertion; ID: insertion/deletion; DD: deletion/deletion; I: insertion; D: deletion; $\chi^{2}$ : chi-squared test; p : probability.

**Table 3:** Polymorphism information of *InDels* in the *YAP/TAZ* gene.

Variety	Site	Number	Population parameter			
			Ho	He	Ne	PIC
SWCG	InDel-1	299	0.858	0.142	1.166	0.132
	InDel-3	299	0.667	0.333	1.499	0.278
	InDel-4	299	0.512	0.488	1.954	0.369
	InDel-5	299	0.566	0.434	1.768	0.340
	InDel-6	299	0.639	0.361	1.564	0.296
	InDel-8	299	0.500	0.500	1.999	0.375
GZDG	InDel-1	266	0.582	0.418	1.717	0.330
	InDel-2	266	0.505	0.495	1.979	0.372
	InDel-7	266	0.501	0.499	1.997	0.375
	InDel-8	266	0.566	0.434	1.766	0.340

SWCG: Shanbei White Cashmere Goat; GZDG: Guanzhong Dairy Goat; Ho: homozygosity; He: heterozygosity; Ne: effective allele number; PIC: polymorphism information content.

### 3.3 Linkage disequilibrium analysis

**Figure 3:**
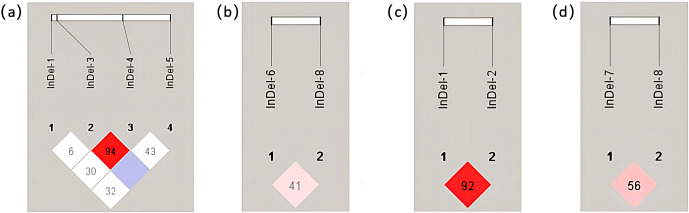
Linkage disequilibrium analysis between different *InDels *loci of the *YAP/TAZ* gene in the two goats. **(a)** Linkage disequilibrium analysis between four *InDels* loci of the *YAP* gene in Shanbei White Cashmere Goat. **(b)** Linkage disequilibrium analysis between four *InDels* loci of the *TAZ* gene in Shanbei White Cashmere Goat. **(c, d)** Linkage disequilibrium analysis between the *YAP* and *TAZ* gene *InDels* loci in Guanzhong Dairy Goat.

The number in the grid was D ${}^{\prime }$ , which was increased by 100 for esthetic reasons. The redder the color of the grid, the stronger the degree of linkage between the two loci, according to linkage disequilibrium analysis performed using the Haploview 4.2 software. In the *YAP* gene of Shanbei White Cashmere Goat, there was greater interlocking between InDel-3 and InDel-4 and less interlocking between the other loci (Fig. 3a). In Shanbei White Cashmere Goat, there was less chaining between the *TAZ* gene's *InDels* sites (Fig. 3b). The degree of interlocking between the InDel-7 and InDel-8 loci of the *TAZ* gene in Guanzhong Dairy Goat decreases with increasing interlocking between the InDel-1 and InDel-2 loci of the *YAP* gene (Fig. 3c, d).

### 3.4 Association analysis of genotype and growth traits

The results of the association analysis between the genotypes of various *InDels* loci and growth traits indicated that, for the growth trait of chest depth, genotype II of InDel-1 was considerably greater than genotype ID ( p<0.01 ). Different genotypes in InDel-3 showed a substantial ( p<0.05 ) correlation with both chest circumference and width. Chest width in InDel-4 was substantially associated ( p<0.05 ) with different genotypes. Different genotypes in InDel-5 showed a substantial ( p<0.05 ) correlation with body height, cross height, and circumference. Table 4 shows a substantial correlation ( p<0.05 ) between different genotypes in InDel-6 and body height as well as a significant correlation ( p<0.0 5) between different genotypes in InDel-8 and waist width.

Table 5 shows that there was a significant correlation between different genotypes of InDel-1, InDel-2, and InDel-7 with respect to body height, rump height, and body length ( p<0.05 ); also, there was a significant correlation between different genotypes of InDel-8 and body weight ( p<0.05 ) and between different genotypes of InDel-1 and rump width, cannon circumference, and body weight ( p<0.01 ).

Surprisingly, across the two goats' various *InDels* loci, nearly all type-II homozygotes had the strongest growth performance (Tables 4 and 5). This showed that the *TAZ* genes InDel-7 and InDel-8 as well as the insertion mutations of the *YAP* genes InDel-1, InDel-2, and InDel-5 were advantageous. Furthermore, the Shanbei White Cashmere Goat *YAP* gene InDel-3 and InDel-4 DD type had the worst growth performance, whereas type-II homozygotes had the best growth performance. These findings suggested that InDel-3 and InDel-4 deletion mutations were detrimental variations. The data that are not significantly different are not provided, and Tables 4 and 5 mostly display the growth performance that differs from the growth features.

**Table 4:** The effect of the *InDels* locus of the *YAP/TAZ* gene on growth traits of Shanbei White Cashmere Goat.

Locus	Growth trait	Genotype (mean ± SE)			p
		II	ID	DD	
InDel-1	ChD (cm)	28.44 ${}^{a}\pm 0.20$ ( n=253 )	26.88 ${}^{b}\pm 0.69$ ( n=46 )	–	0.006
InDel-3	ChW (cm)	19.09 ${}^{a}\pm 0.23$ ( n=181 )	17.95 ${}^{b}\pm 0.31$ ( n=110 )	16.59 ${}^{b}\pm 0.82$ ( n=8 )	0.002
	ChC (cm)	82.55 ${}^{a}\pm 0.57$ ( n=181 )	79.96 ${}^{b}\pm 1.08$ ( n=110 )	77.81 ${}^{\mathrm{ab}}\pm 3.13$ ( n=8 )	0.038
InDel-4	ChW (cm)	19.18 ${}^{a}\pm 0.33$ ( n=93 )	18.56 ${}^{a}\pm 0.25$ ( n=160 )	17.60 ${}^{b}\pm 0.44$ ( n=46 )	0.020
InDel-5	BH (cm)	59.32 ${}^{a}\pm 0.97$ ( n=29 )	56.61 ${}^{b}\pm 0.42$ ( n=133 )	56.87 ${}^{b}\pm 0.42$ ( n=137 )	0.025
	HHC (cm)	62.53 ${}^{a}\pm 1.0$ 3 ( n=29 )	59.53 ${}^{b}\pm 0.4$ 3 ( n=133 )	60.32 ${}^{b}\pm 0.4$ 4 ( n=137 )	0.015
	CaC (cm)	8.82 ${}^{a,b}\pm 0.78$ ( n=29 )	8.58 ${}^{b}\pm 0.80$ ( n=133 )	8.86 ${}^{a}\pm 0.90$ ( n=137 )	0.026
InDel-6	BH (cm)	57.58 ${}^{a}\pm 0.37$ ( n=173 )	56.07 ${}^{b}\pm 0.46$ ( n=111 )	57.11 ${}^{a}\pm 1.36$ ( n=15 )	0.042
InDel-8	WAW (cm)	18.83 ${}^{a}\pm 0.3$ 6 ( n=73 )	19.44 ${}^{a}\pm 0.2$ 2 ( n=148 )	18.39 ${}^{b}\pm 0.2$ 8 ( n=78 )	0.019

ChD: chest depth; ChW: chest width; ChC: chest circumference; BH: body height; HHC: height at hip cross; CaC: cannon circumference; WAW: waist angle width; SE: standard error; II: insertion/insertion; ID: insertion/deletion; DD: deletion/deletion. ${}^{a,b}$ Values within a row with different superscripts differ significantly at p<0.05 .

**Table 5:** Effects of the *InDels* locus of the *YAP/TAZ* gene on growth traits of Guanzhong Dairy Goat.

Locus	Growth trait	Genotype (mean ± SE)			p
		II	ID	DD	
InDel-1	ChC (cm)	102.57 ${}^{a}\pm 2.27$ ( n=28 )	91.33 ${}^{b}\pm 2.23$ ( n=102 )	88.35 ${}^{b}\pm 0.64$ ( n=136 )	0.001
	CaC (cm)	11.29 ${}^{a}\pm 0.42$ ( n=28 )	8.80 ${}^{b}\pm 0.43$ ( n=102 )	7.97 ${}^{b}\pm 0.03$ ( n=136 )	0.001
	TW (cm)	19.00 ${}^{a}\pm 0.49$ ( n=28 )	17.47 ${}^{a,b}\pm 0.55$ ( n=102 )	16.77 ${}^{b}\pm 0.09$ ( n=136 )	0.001
	BW (kg)	76.00 ${}^{b}\pm 0.37$ ( n=28 )	76.57 ${}^{b}\pm 0.24$ ( n=102 )	77.59 ${}^{a}\pm 0.24$ ( n=136 )	0.001
InDel-2	ChW (cm)	96.50 ${}^{a}\pm 2.26$ ( n=84 )	90.17 a±1.42 ( n=125 )	87.63 ${}^{b}\pm 0.96$ ( n=57 )	0.002
	CaC (cm)	9.93 ${}^{a}\pm 0.51$ ( n=84 )	8.30 ${}^{b}\pm 0.25$ ( n=125 )	8.00 ${}^{b}\pm 0.00$ ( n=57 )	0.001
	RW (cm)	18.00 ${}^{a}\pm 0.41$ ( n=84 )	17.22 ${}^{a,b}\pm 0.36$ ( n=125 )	16.69 ${}^{b}\pm 0.1$ 2 ( n=57 )	0.044
	BW (kg)	76.34 ${}^{b}\pm 0.24$ ( n=84 )	76.92 ${}^{b}\pm 0.26$ ( n=125 )	78.09 ${}^{a}\pm 0.31$ ( n=57 )	0.001
InDel-7	BH (cm)	75.78 ${}^{a}\pm 1.00$ ( n=60 )	72.98 ${}^{b}\pm 0.52$ ( n=136 )	72.47 ${}^{b}\pm 0.83$ ( n=70 )	0.010
	RH (cm)	76.52 ${}^{a}\pm 0.99$ ( n=60 )	73.65 ${}^{b}\pm 0.50$ ( n=136 )	73.10 ${}^{b}\pm 0.84$ ( n=70 )	0.007
	TL (cm)	77.39 ${}^{a}\pm 1.15$ ( n=60 )	74.67 ${}^{b}\pm 0.50$ ( n=136 )	74.59 ${}^{b}\pm 0.79$ ( n=70 )	0.026
InDel-8	BW (kg)	77.89 ${}^{a}\pm 0.2$ 2 ( n=144 )	76.16 ${}^{b}\pm 0.2$ 1 ( n=102 )	76.67 ${}^{b}\pm 0.6$ 2 ( n=20 )	0.001

ChC: chest circumference; CaC: cannon circumference; TW: thurl width; BW: body weight; ChW: chest width; RW: rump width; BH: body height; RH: rump height; TL: trunk length; SE: standard error; II: insertion/insertion; ID: insertion/deletion; DD: deletion/deletion. ${}^{a,b}$ Values within a row with different superscripts differ significantly at p <0.05.

## 4 Discussion

One of the main causes of genetic diversity is insertion and deletion mutation. Animal growth and development may be impacted by *InDels* mutations in the genome due to a variety of phenotypic and molecular effects (Pagel et al., 2019). In this study, three *InDels* were discovered in the *TAZ* gene's intron area, and five *InDels* were discovered in the *YAP* gene's intron in two local goat breeds. Table 2 shows that most polymorphism information content was moderately polymorphic, and the highest population heterozygosity (0.418–0.500) and effective allele count (1.717–1.999) were found in InDel-4, InDel-5, and InDel-8 of Shanbei White Cashmere Goat and InDel-1, InDel-7, and InDel-8 of Guanzhong Dairy Goat, respectively. These results suggest that the population has more evenly distributed alleles of these *InDels* loci, the highest degree of genetic variation, and a richer genetic diversity overall. Furthermore, the frequency of the D allele is low, and there is no DD genotype in InDel-1 of Shanbei White Cashmere Goat. Either too little sample size or homozygous embryo mortality could be the cause of this.

According to Crow (1988), the Hardy–Weinberg law is the basis of contemporary genetics and the basis of population genetics. HWE is able to determine the reliability of population survey data in addition to estimating gene frequency from phenotype and genotype frequency in terms of population genetics of human genetic markers. The deviation from the Hardy–Weinberg equilibrium happens when there are selection, drift, population mixing, or non-random mating forms (Kang and Shin, 2004; Wigginton et al., 2005). All of the *InDels* loci of the *YAP/TAZ* gene were in Hardy–Weinberg equilibrium, according to the study's $\chi^{2}$ test results (Table 2).

Numerous studies have suggested that variations in *InDels* may impact an animal's growth qualities, and *InDels* are frequently employed in molecular marker-assisted breeding as a molecular marker linked to significant economic attributes (Wu et al., 2020). For example, Wang et al. (2020) and Zhang et al. (2021) found a substantial correlation between litter size and the 21 bp InDel mutation in the *ATBF1* gene and the 16 bp InDel mutation in the *AKAP13* gene. Male sheep's tail fat deposition was considerably impacted by the *FTO* gene's *InDels* mutation (Wang et al., 2021). Chest depth in Lanzhou big-tailed sheep and body weight, body height, and other body-measuring features in Ruxi black-headed sheep were strongly impacted by the 7 bp InDel locus in the *KDM3B* gene (Kang et al., 2022). In addition, the Del/Del genotype of the rs669452874 locus of the *GLIPR1L1 *gene significantly affected the height and length of Dazu black goats, Hechuan white goats, and Inner Mongolian Cashmere goats (Saleh et al., 2023).

According to Shaul (2017), introns can boost mRNA translation efficiency and stability in the cytoplasm. Enhancers and silencers are examples of cis-acting regions in introns that influence mRNA shearing. Additionally, intron mutations may cause mRNAs to detect incorrect sequences during splicing, resulting in improper removal of introns, which may alter open reading frames (Sarkar, 2022). The *YAP* gene's introns containing the mutations of InDel-1, InDel-2, InDel-3, InDel-4, and InDel-5 in this study had a substantial impact on the goats' body height, cross height, cannon circumference, rump breadth, and body weight. The goats' body height, rump height, body length, and body weight were all positively impacted by the mutations of InDel-6, InDel-7, and InDel-8 in the *TAZ* gene's introns. Important osteogenesis mediators include *TAZ* and *YAP*. According to Zaidi et al. (2004), *YAP* can block the Runx2-dependent transcriptional activation of osteocalcin (OCN). Furthermore, osteogenesis is sped up by *YAP* deficiency and inhibited by high expression of *YAP *in bone marrow mesenchymal stem cells. According to studies, *YAP* prevents endochondral ossification during the growth and healing of bones. Defects in bone formation occur in vivo when the *YAP/TAZ* combination is absent (Yang et al., 2019). The Hippo signaling pathway plays an important role in the control of organ size in animals, and *YAP* is a transcriptional co-activator and a key effector in the Hippo signaling pathway. The results of Ishihara and Nishina (2018) showed that *YAP/TAZ* regulates organ size by modulating cellular tone: in addition, in a chimeric mouse model, hepatocytes with impaired *YAP *gene expression activity undergo apoptosis and are cleared from the liver. Therefore, *YAP/TAZ* has the function of controlling the number and quality of cells in the process of organogenesis, and the organ size is closely related to the body size of animals. Thus, *YAP/TAZ* affects the body size of animals by influencing the organ size. Situated at the core of an intricate signal network with multiple biological purposes, *YAP/TAZ* is crucial for controlling animal growth.

Briefly, to the best of our knowledge, there is a wide range of differences for body measurements and the body indices among breeds of goats (Saleh et al., 2022). The screening and function identification of *InDel* markers is beneficial for further developing and utilizing high-quality genes and germplasm resources.

## 5 Conclusions

This investigation identified *YAP/TAZ* polymorphisms from northern Shanbei White Cashmere Goat and Guanzhong Dairy Goat, determined the candidate *InDels*, and uncovered their association with phenotypic data of the productive performance. These *InDels* loci may provide a database for genetic analysis and molecular marker-assisted breeding in goat populations. The economic efficiency of the goat industry can be increased by applying these insights to the breeding process.

## Data Availability

Animal data were not deposited in an official repository and are confidential.

## References

[bib1.bib1] Barrett JC (2009). Haploview: Visualization and analysis of SNP genotype data. Cold Spring Harbor Protocols.

[bib1.bib2] Botstein D, White RL, Skolnick M, Davis RW (1980). Construction of a genetic linkage map in man using restriction fragment length polymorphisms. Am J Hum Genet.

[bib1.bib3] Crow JF (1988). Eighty years ago: the beginnings of population genetics. Genetics.

[bib1.bib4] Cui Y, Yan H, Wang K, Xu H, Zhang X, Zhu H, Liu J, Qu L, Lan X, Pan C (2018). Insertion/Deletion Within the KDM6A Gene Is Significantly Associated With Litter Size in Goat. Front Genet.

[bib1.bib5] Fu W, Wang R, Yu J, Hu D, Cai Y, Shao J, Jiang Y (2021). GGVD: A goat genome variation database for tracking the dynamic evolutionary process of selective signatures and ancient introgressions. J Genet Genomics.

[bib1.bib6] Heng BC, Zhang X, Aubel D, Bai Y, Li X, Wei Y, Fussenegger M, Deng X (2021). An overview of signaling pathways regulating YAP/TAZ activity. Cell Mol Life Sci.

[bib1.bib7] Ishihara E, Nishina H (2018). The Hippo-YAP Pathway Regulates 3D Organ Formation and Homeostasis. Cancers.

[bib1.bib8] Kang SH, Shin D (2004). The size of the chi-square test for the Hardy-Weinberg law. Hum Hered.

[bib1.bib9] Kang Y, Bi Y, Tang Q, Xu H, Lan X, Zhang Q, Pan C (2022). A 7-nt nucleotide sequence variant within the sheep KDM3B gene affects female reproduction traits. Anim Biotechnol.

[bib1.bib10] Li X, Wu Q, Zhang X, Li C, Zhang D, Li G, Zhang Y, Zhao Y, Shi Z, Wang W, Li F (2021). Whole-Genome Resequencing to Study Brucellosis Susceptibility in Sheep. Front Genet.

[bib1.bib11] Li Y, Yang S, Qin L, Yang S (2021). TAZ is required for chondrogenesis and skeletal development. Cell Discovery.

[bib1.bib12] Lin W, Ren T, Li W, Liu M, He D, Liang S, Luo W, Zhang X (2021). Novel 61-bp InDel of RIN2 Is Associated With Fat and Hatching Weight Traits in Chickens. Front Genet.

[bib1.bib13] Liu YQ, Chen MJ, Wang YJ, Xu HF, Li M, Yu GQ (2023). YAP1 Regulating the Muscle Growth and Development in New ZealandWhite Rabbits. Chinese Journal of Biochemistry and Molecular Biology.

[bib1.bib14] Matin A, Nadeau JH (2001). Sensitized polygenic trait analysis. Trends Genet.

[bib1.bib15] Nei M, Li WH (1979). Mathematical model for studying genetic variation in terms of restriction endonucleases. P Natl Acad Sci USA.

[bib1.bib16] Nei M, Roychoudhury AK (1974). Sampling variances of heterozygosity and genetic distance. Genetics.

[bib1.bib17] Pagel KA, Antaki D, Lian A, Mort M, Cooper DN, Sebat J, Iakoucheva LM, Mooney SD, Radivojac P (2019). Pathogenicity and functional impact of non-frameshifting insertion/deletion variation in the human genome. PLoS Comput Biol.

[bib1.bib18] Piccolo S, Dupont S, Cordenonsi M (2014). The biology of YAP/TAZ: hippo signaling and beyond. Physiol Rev.

[bib1.bib19] Pocaterra A, Romani P, Dupont S (2020). YAP/TAZ functions and their regulation at a glance. J Cell Sci.

[bib1.bib20] Saleh AA, Rashad AMA, Hassanine NNAM, Sharaby MA, Zhao Y (2022). Morphological body measurements, body indices, and their genetic background for several Chinese goat breeds. Trop Anim Health Pro.

[bib1.bib21] Saleh AA, Xue L, Zhao Y (2023). Screening InDels from the whole genome to identify the candidates and their association with economic traits in several goat breeds. Funct Integr Genomic.

[bib1.bib22] Sarkar A, Panati K, Narala VR (2022). Code inside the codon: The role of synonymous mutations in regulating splicing machinery and its impact on disease. Mutation research, Reviews in Mutation Research.

[bib1.bib23] Shaul O (2017). How introns enhance gene expression. Int J Biochem Cell Biol.

[bib1.bib24] Wang K, Hui Y, Zhang S, Wang M, Yan H, Zhu H, Qu L, Lan X, Pan C (2020). A deletion mutation within the ATBF1 gene is strongly associated with goat litter size. Anim Biotechnol.

[bib1.bib25] Wang S, Liu S, Yuan T, Sun X (2021). Genetic effects of FTO gene insertion/deletion (InDel) on fat-tail measurements and growth traits in Tong sheep. Anim Biotechnol.

[bib1.bib26] Wigginton JE, Cutler DJ, Abecasis GR (2005). A note on exact tests of Hardy-Weinberg equilibrium. Am J Hum Genet.

[bib1.bib27] Wu M, Zhao H, Tang X, Li Q, Yi X, Liu S, Sun X (2020). Novel InDels of GHR, GHRH, GHRHR and Their Association with Growth Traits in Seven Chinese Sheep Breeds. Animals.

[bib1.bib28] Xiong J, Almeida M, O'Brien CA (2018). The YAP/TAZ transcriptional co-activators have opposing effects at different stages of osteoblast differentiation. Bone.

[bib1.bib29] Yan Y, Yi G, Sun C, Qu L, Yang N (2014). Genome-wide characterization of insertion and deletion variation in chicken using next generation sequencing. PloS one.

[bib1.bib30] Yang B, Sun H, Chen P, Fan N, Zhong H, Liu X, Wu Y, Wang J (2019). YAP1 influences differentiation of osteoblastic MC3T3-E1 cells through the regulation of ID1. J Cell Physiol.

[bib1.bib31] Zaidi SK, Sullivan AJ, Medina R, Ito Y, van Wijnen AJ, Stein JL, Lian JB, Stein GS (2004). Tyrosine phosphorylation controls Runx2-mediated subnuclear targeting of YAP to repress transcription. EMBO J.

[bib1.bib32] Zarka M, Haÿ E, Cohen-Solal M (2022). YAP/TAZ in Bone and Cartilage Biology. Front Cell Dev Biol.

[bib1.bib33] Zhang X, Yuan R, Bai Y, Yang Y, Song X, Lan X, Pan C (2021). A deletion mutation within the goat AKAP13 gene is significantly associated with litter size. Anim Biotechnol.

